# SLC11A1 as a stratification indicator for immunotherapy or chemotherapy in patients with glioma

**DOI:** 10.3389/fimmu.2022.980378

**Published:** 2022-11-30

**Authors:** Houshi Xu, Anke Zhang, Chaoyou Fang, Qingwei Zhu, Wei Wang, Yibo Liu, Zeyu Zhang, Xiaoyu Wang, Ling Yuan, Yuanzhi Xu, Anwen Shao, Meiqing Lou

**Affiliations:** ^1^ Department of Neurosurgery, Shanghai General Hospital, Shanghai Jiao Tong University School of Medicine, Shanghai, China; ^2^ Department of Neurosurgery, Huashan Hospital, Shanghai Medical College, Fudan University, Shanghai, China; ^3^ Department of Neurosurgery, Peking Union Medical College Hospital, Chinese Academy of Medical Sciences and Peking Union Medical College, Beijing, China; ^4^ Department of Neurosurgery, Second Affiliated Hospital, School of Medicine, Zhejiang University, Zhejiang, China; ^5^ Department of Urology, The Second Hospital of Shanxi Medical University, Taiyuan, Shanxi, China

**Keywords:** glioma, immunotherapy, biomarker, immune infiltration, prognosis

## Abstract

**Background:**

Glioma is a fatal tumor originating from the brain, which accounts for most intracranial malignancies. Currently, Immunotherapy has turned into a novel and promising treatment in glioma patients. however, there are still few effective biomarkers to mirror the reaction to immunotherapy in patients with glioma. Therefore, we intended to elucidate the evaluable efficacy of SLC11A1 in glioma patients.

**Methods:**

In this study, samples from Shanghai General Hospital and data from TCGA, GEO, CGGA datasets were used to investigate and validate the relationship between SLC11A1 and the progression of glioma. We evaluated the predictive value of SLC11A1 on the prognosis of glioma with cox regression analysis. Then the relationship between immune infiltration and SLC11A1 was also analyzed. Ultimately, we performed the prediction on the immunotherapeutic response and therapeutic drugs according to the expression of SLC11A1.

**Results:**

Expression of SLC11A1 increased with progression and predicted unfavorable prognosis for glioma patients. The hazard ratio for SLC11A1 expression was 2.33 with 95% CI (1.92-2.58) (P < 0.001) in cox analysis. And based on expression, we found SLC11A1 stratified glioma patients into subgroups with different immune activation statuses. Moreover, we observed that patients with higher SLC11A1 levels companied with better immunotherapeutic response, while those with lower SLC11A1 levels may respond better to temozolomide.

**Conclusion:**

This study provided evidence that SLC11A1 was a novel prognostic marker and immunotherapy response indicator for gliomas. In some cases, SLC11A1 could be an effective marker for identifying patients who might benefit from immunotherapy or chemotherapy.

## Introduction

Glioma, the most common primary brain tumor, companied with poor prognosis in human adult (1). It is a type of rapidly progressing tumor, and the overall survival time in newly diagnosed glioma patients is approximately 12–18 months. Despite a variety of therapeutic approaches for gliomas, the outcome for patients with glioma is still poor. Surgery is not effective due to the tumor’s infiltrative nature. Due to tumor heterogeneity and epigenetic complexity, it is difficult to identify therapeutic targets for glioma. Additionally, the delivery of chemotherapeutic drugs is limited by the blood-brain barrier (BBB). Therefore, a thorough understanding of the factors involved in tumor progression is critical to exploring effective strategies for the diagnosis and treatment of glioma patients.

The use of cancer immunotherapy, such as immune checkpoint blockade (ICB), has been proven to benefit patients with gliomas. ICB inhibits tumor progression through the reinvigoration of tumor cytotoxic T cells. Although glioma is an immunogenic tumor characterized by high neoantigen levels, only a small subset of patients responds to ICB due to primary or secondary drug resistance (2). In light of the significant economic burden and side effects associated with radiochemotherapy, it is necessary to explore more robust predictive biomarkers for ICB response (3). However, because of complicated molecular methods, the detection of biomarkers tends to be expensive. Although several molecular biomarkers have been identified to predict the prognosis and therapeutic response to glioma, further study is needed to facilitate their widespread clinical application. Thus, there is an urgent medical need for fast and economical molecular subtype predictors.

A recent study showed that solute carrier family 11 member 1 (SLC11A1) has multiple effects on macrophage activation and exerts a vital role in immune response (4). Susceptibility to infections and autoimmune diseases is linked to SLC11A1. Moreover, SLC11A1 has been proved a correlation with various tumors, such as bladder cancer and esophageal cancer (5, 6). SLC11A1 expression has been implicated in bladder cancer recurrence and the response to Calmette–Guerin (BCG) immunotherapy (5). However, no study has revealed the function of SLC11A1 in the development of glioma, and the potential molecular mechanism is poorly understood.

Our study examined the expression patterns of SLC11A1 and its immunological function across a range of cancers. Anti-SLC11A1 therapy appears to be an appropriate treatment for gliomas. We also report that SLC11A1 expression promotes tumor progression and may serve as a biomarker for differentiating molecular subtypes of gliomas.

## Materials and methods

### Tumor samples collection

Human samples were exempt from testing by Shanghai General Hospital’s Human Investigation Ethical Committee. The samples were recruited between January 2021 and January 2022 from the Department of Neurosurgery in Shanghai General Hospital. Among the 20 glioma patients (Grade II: n = 7; III: n = 6; IV: n = 7) none had experienced chemotherapy and radiotherapy before. Each patient signed an informed agreement paper.

### Data source and expression analysis

A pan-cancer dataset from The Cancer Genome Atlas (TCGA) was analyzed with UCSCXenaShiny containing 33 subtypes of cancer and GTEx expression matrix data (7). All data on gliomas came from Gliovis (8) (**Supplementary Table 1**). Single cell RNA sequencing of gliomas was derived from GEO database (GSE131928) (9) with 7930 high quality cells acquired from 28 patients. Single cell data analysis was carried out by *Seurat4.0* (10). Cell annotation was performed with R package “SingleR” (11) and markers derived from TISCH (12). Spatial transcriptome data of glioma was downloaded from 10X genomics main page(https://www.10xgenomics.com/cn/resources/datasets/human-glioblastoma-whole-transcriptome-analysis-1-standard-1-2-0). After imported into R, the filtered UMI count matrix was analyzed using the R package Seurat (10). Then we used regularized negative binomial regression (SCTransform) to normalize UMI count matrices. Top 3,000 highly variable genes were identified. We determined to use the first 30 principal components in clustering analysis. UMAP dimensionality reduction was performed with the first 30 principal components as input to visualize spots. SpatialFeaturePlot() function was used for gene expression in spots.

### Immunohistochemical and immunofluorescence analysis

Paraformaldehyde 4% and paraffin were added to the samples to fix them for 24 hours. After cutting the paraffin block into five millimeter-thick sections then blocked overnight at 4°C and stained with SLC11A1 (Abcam, ab211448, USA). Using biotinylated rabbit IgG incubated with PBS after washing the sections. The sections were viewed under a microscope AX-80. The images were analyzed by Image J.

Sections that were formalin-fixed and embedded in paraffin were deparaffinized, rehydrated, permeabilized, and rinsed. Antigen repair in citrate buffer was made for 15 min. Blocking was carried out in 5% BSA for 1 h at room temperature. Then sections were stained with SLC11A1 (Lifespan, LS-B9344, USA), CD68 (Abcam, ab201340, USA), PD1 (Abcam, ab52587, USA). 2% BSA/PBS was diluted in secondary antibodies, which were incubated for another 1 h. After stain with DAPI, microscope images were taken of the sections.

### Real-time PCR

Total RNA was extracted from human sample using TRIzol reagent. The reverse transcription is performed with FastQuant RT kit. Real-time PCR was carried out using SuperReal SYBR Green kit in Lightcycler 96). The primer sequences were listed as follow: SLC11A1 forward: GCACCTCCCAGAGAGACCT; reverse: GAGCAGCACCCAGAGAAGTT; PDCD1 forward: CAGTTCCAAACCCTGGTGGT; reverse: GGCTCCTATTGTCCCTCGTG; CA4 forward: CAAGTGCCTTCTGTGTGTGC; reverse: GAGCGGTGTTCAGGTCTTCA.

### Bioinformatic analysis

TCGA mutation data was derived from R package “*TCGAmutations*” (13) (study=“GBM” and “LGG”), data analysis was performed by “*maftools*” (14). The raw mutation count for TMB (Tumor Mutation Burden) analysis was determined by TCGA using the somatic variants. An estimated size of 38 Mb was used for the exome. On the basis of the level of SLC11A1, glioma patients from CGGA dataset were grouped into high group and low expression group. Differential expressed gene (DEGs) analysis were performed by R package “*limma*” (15). The biological significance of the DEGs was defined as |logFC|≥1.5 and adj.pvalue <0.05 (16).

Using the R package “Pi” we further investigated the functional enrichment with Gene Set Enrichment Analysis (GSEA) (17). In order to explore the association between SLC11A1 expression and immune status, 25 immune-related genes were analyzed based on a previous study (18) (**Supplementary Table 2**). To landscape the immune profile of glioma samples, we performed Gene Set Variation Analysis using the R package “GSVA”.

### Quantify of relative abundance of immune cells and prediction of the immunotherapy response

ssGSEA was used to calculate a enrichment score indicating how much a gene set was enriched in each sample of a dataset with R package “*GSVA*” (19). According to a previous study, we obtained 28 types of immune cells’ gene set signatures (20). To prevent bias caused by a singular algorithm, other methods for calculating the relative abundance of cells in the immune microenvironment were also used: Cibersort-ABS (21), MCP-counter (22), quanTIseq (23), TIMER (24) and xCell (25), the immune cells data were downloaded from TIMER (http://timer.cistrome.org/). Expression and survival data from CGGA were merged and MCP-counter variables together with SLC11A1 were binarized using a median cut (leading to “high” and “low” samples for each variable from the cell’s median value or gene expression). For this study, we concatenated the binarized scores for the two variables of interest (Mono/Macro cells and SLC11A1), leading to four classes (high–high, high–low, low–high, low–low). The corresponding Kaplan–Meier curves for OS were then plotted and the p value of the corresponding log-rank test is calculated. This algorithm was previously described in Etienne et al’s study (22).

To anticipate their response to anti-PDL1 drug, the GSVA method using the T-cell inflammatory (TIS) signature were used to score the glioma samples. This signature was listed in **Supplementary Table 3**. Immune Cell Abundance Identifier (ImmuCellAI) (26) and Tumor Immune Dysfunction and Exclusion (TIDE) (27) were performed to investigate the potential response of ICB therapy. The Subclass Mapping (SubMap) method was also used to evaluate the role of SLC11A1in 47 patients with different immunotherapy responses (28).

### Drug sensitivity analysis

Drug sensitivity data of CCLs were acquired from the Cancer Therapeutics Response Portal (CTRP v.2.0) and PRISM Repurposing dataset (19Q4). The *pRRophetic* package (29) which had a built-in ridge regression model was used to predict the drug response. CTRP and PRISM dataset each provides the area under the dose–response curve values as a measure of drug sensitivity.

### Statistical analysis

R software 4.0.5 was used to perform all statistical analysis. We used the Kolmogorov-Smirnov test to identify whether a non-parametric or a parametric analysis should be applied to every dataset based on the distribution normality. The correlation analysis was made based on Spearman correlation analysis. Fisher exact test and Wilcoxon rank-sum tests were performed to compare categorical and continuous variables. By using Cox proportional hazard models, survival analysis assessed the association between overall survival and characteristics. Kaplan-Meier survival curves were drawn and compared with R packages “*survival*” and “*survminer*”. Meta-analysis was performed with R package “*meta*” (30). P value < 0.05 was considered significant. Oncoplot was generated with R package “*ComplexHeatmap*” (31). Fishplot was plotted using “ *fishplot*” (32) package.

## Result

### Overview of *SLC11A1* in various tumors

**Figure 1:**
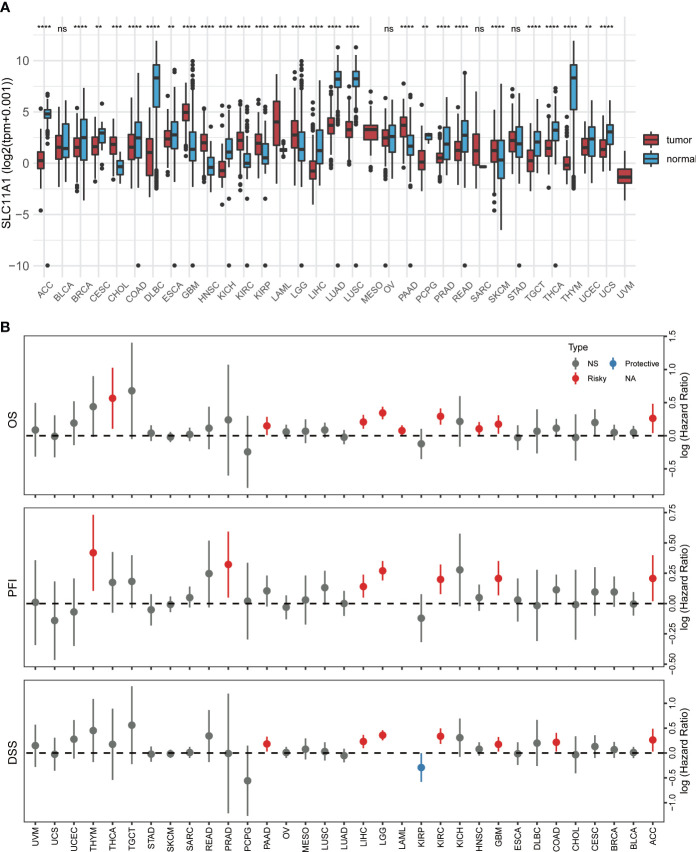
Pan-cancer analysis of SLC11A1 expression. **(A)** UCSCXenaShiny was used to visualize SLC11A1 expression in the cancer genome atlas (TCGA) pan-cancer datasets. **(B)** Risk plot of correlation SLC11A1 with OS, PFI, DSS (red represents HR > 1(risky) and P value < 0.05; blue represents HR < 1 (protective) and P value < 0.05; grey represents no statistical significance). **,P < 0.01; ***,P < 0.001; ****,P < 0.0001, ns = no significance (Wilcoxon test).

To investigate the clinical value of SLC11A1 expression in human tumors, the levels of SLC11A1 expression in normal tissues and tumor samples based on TCGA and GTEx databases were analyzed using UCSCXenaShiny (7). As shown in **Figure 1A**, SLC11A1 was significantly upregulated in several cancer tissues compared with normal tissues (all p < 0.05), and was downregulated in several other cancer tissues. The results suggested that SLC11A1 exerts different roles in tumors. Further, we aimed to determine whether SLC11A1 levels are associated with clinical outcomes in patients with different cancers. We utilized UCSCXenaShiny to assess the role of SLC11A1 expression on outcome based on univariate Cox analysis. Based on the expression of SLC11A1, patients were split into two SLC11A1 subgroups. The results suggested that high SLC11A1 expression was associated with poor outcomes in ACC, KIRC, LGG, GBM, LIHC, LAML, PRAD, PAAD, and THYM. Above results showed that SLC11A1 was predominantly correlated with a poor prognosis in patients with human tumors, especially glioma (**Figure 1B**).

### The elevation of *SLC11A1* expression indicates poor clinical outcomes in patients with glioma

**Figure 2:**
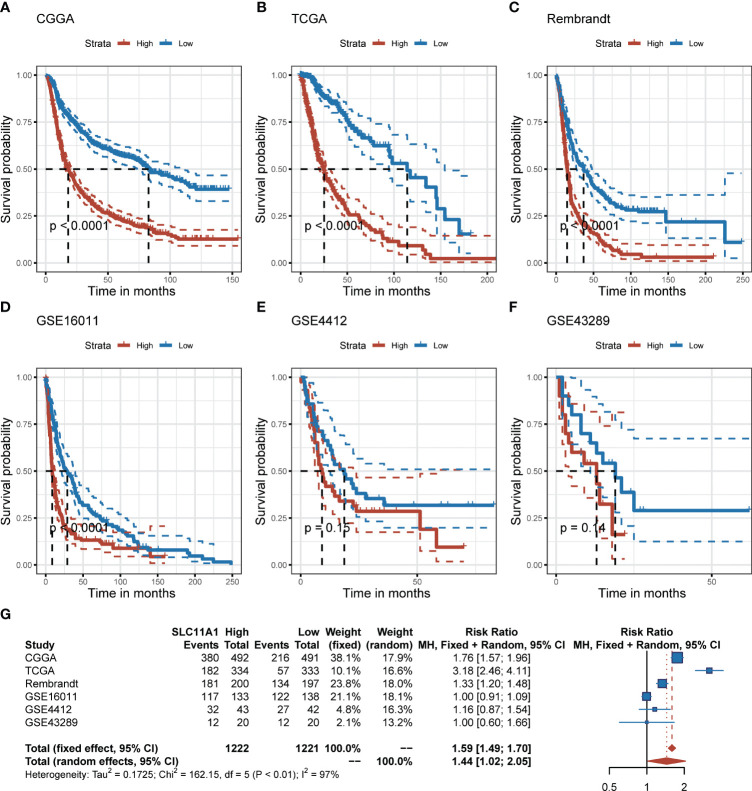
Elevation of SLC11A1 expression predicts poor prognosis in gliomas. Kaplan-Meier plots of SLC11A1 in six glioma datasets, 95% CI was also showed. Patients were divided into high and low expressed group by the medium expression level. **(A)** CGGA, **(B)** TCGA, **(C)** Rembrandt, **(D)** GSE16011, **(E)** GSE4412, and **(F)** GSE43289. **(G)** Forest plot of the RRs for patients with high SLC11A1 expression compared to patients with low SLC11A1 expression.

To further explore the effect of SLC11A1 in gliomas, we studied the association between its expression and prognosis of gliomas through the analysis of six datasets (n=2390). Based on the expression of SLC11A1, patients were classified into high-SLC11A1 or low-SLC11A1 subgroup. Then the log-rank test analysis indicated that patients with high expression of SLC11A1 in the Chinese Glioma Genome Atlas (CGGA), TCGA, Rembrandt and GSE16011 cohorts presented markedly poorer prognoses than those with low expression of SLC11A1 (**Figures 2A–D**), while a similar but nonsignificant trend was observed in patients derived from the GSE4412 and GSE43289 cohorts (**Figures 2E, F**). As shown in **Figure 2G**, there was a shorter overall survival time for patients whose SLC11A1 expression was high compared to patients whose expression was low (RR = 1.59, 95% CI 1.49-1.70).

**Figure 3:**
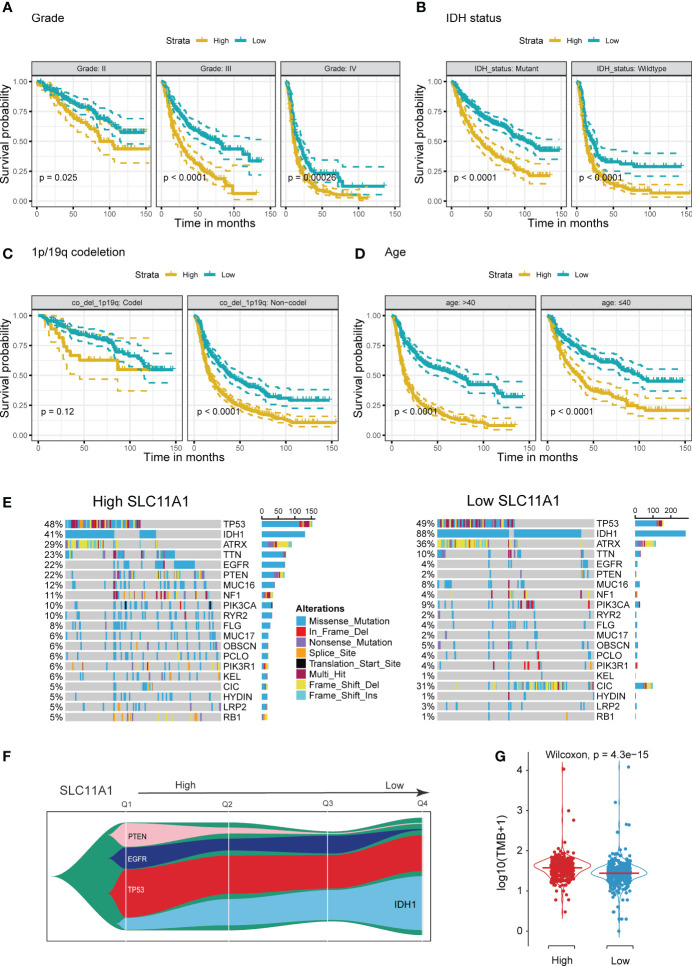
Stratification Analysis and Mutation landscape of high/low SLC11A1 subgroups. High SLC11A1 expression predicts poor prognosis in gliomas with different clinical characteristics. Patients were divided into high and low expressed group by the medium expression level. Kaplan-Meier plots of SLC11A1 were performed with a variety of clinical characteristics. **(A)** tumor grade, **(B)** IDH mutational status, **(C)** 1p/19q co-deletion, and **(D)** Age. **(E)** Oncoplots showed the top 20 genes of mutations in patients with high expression of SLC11A1. **(F)** Fishplot showed that with SLC11A1’s expression changing from high to low, IDH1 mutations progressively account for the dominant type of total mutations. (Q: quartile) **(G)** Violin plot showed higher tumor mutation burdens in patients with high SLC11A1 expression compared to those with low SLC11A1 expression.

Using the TCGA cohort, we performed subgroup analyses to determine if SLC11A1 is associated with positive prognosis in different subgroups of glioma patients. For high grade gliomas, a low expression level of SLC11A1 indicated a better prognosis (p < 0.05) (**Figure 3A**). Similarly, in isocitrate dehydrogenase (IDH)-mutant or wild-type glioma patients, longer survival times exist in the low SLC11A1 group (p < 0.05) (**Figure 3B**). Moreover, among those with 1p/19q codeletion and non-codeletion subtypes, the prognosis was extremely different (codel: p=0.12; non-codel: p<0.05) (**Figure 3C**), which was in accordance with the results in the young (age ≤40 years old) vs. old (age >40 years old) groups (**Figure 3D**).

IDH mutation usually indicates a good prognosis in glioma patients. We obtained mutation data from the TCGA dataset. According to the gene expression, patients were categorized into two subgroups, and the patients with a higher SLC11A1 expression had a lower rate of IDH mutation and higher rates of EGFR and PTEN mutations (**Figures 3E, F** and **Supplementary Figure 1A**), and which are considered to indicate a poor prognosis for glioma patients. Glioma patients with wild-type IDH1, mutant EGFR or mutant PTEN showed higher expression of SLC11A1 (**Supplementary Figure 1B**) than those with other phenotypes. Additionally, tumor mutation burden (TMB) analysis revealed that low SLC11A1 expression usually accompanied by a lower TMB, and a key role for TMB is considered in the generation of immunogenic neopeptides displayed on tumor cells is its role in driving the expression of MHC molecules on tumor cells (**Figure 3G**). The above results indicate that SLC11A1 is a potential novel biomarker for predicting survival of patients.

### The level of *SLC11A1* expression increased with the malignancy of gliomas

To further determine the clinical significance of SLC11A1 in glioma patients, the clinical study data of 1018 patients with glioma obtained from the CGGA dataset was included to analyzed. According to the expression of SLC11A1, patients were split into two different group (509 vs 509). Statistical analysis showed that high SLC11A1 expression related to older age, shorter survival time, higher tumor grade, GBM subtype, mesenchymal subtype, and wild type IDH, which further confirmed the findings from the TCGA analysis (**Table 1**).

**Table 1:** Clinical characteristics of 1018 glioma patients in the CGGA dataset according to SLC11A1 expression levels.

Variable	n	Overall, n = 1,018^1^	High, n = 509^1^		Low, n = 509^1^	p-value^2^
**Age**	1,017	43 (35, 51)	45 (37, 55)	41 (34, 48)		<0.001
**Gender**	1,018					0.3
Female		422 (41%)	202 (40%)	220 (43%)		
Male		596 (59%)	307 (60%)	289 (57%)		
**survival**	983	39 (11, 59)	29 (8, 40)	48 (17, 76)		<0.001
**status**	989					<0.001
Alive		388 (39%)	113 (23%)	275 (56%)		
Dead		601 (61%)	382 (77%)	219 (44%)		
**Grade**	1,013					<0.001
II		291 (29%)	92 (18%)	199 (39%)		
III		334 (33%)	143 (28%)	191 (38%)		
IV		388 (38%)	271 (54%)	117 (23%)		
**Histology**	1,013					
Oligodendroglioma		112 (11%)	11 (2.2%)	101 (20%)		
Oligoastrocytoma		9 (0.9%)	3 (0.6%)	6 (1.2%)		
Astrocytoma		175 (17%)	79 (16%)	96 (19%)		
Anaplastic Oligodendrolgioma		94 (9.3%)	16 (3.2%)	78 (15%)		
Anaplastic Oligoastrocytoma		21 (2.1%)	10 (2.0%)	11 (2.2%)		
Anaplastic Astrocytoma		214 (21%)	116 (23%)	98 (19%)		
GBM		388 (38%)	271 (54%)	117 (23%)		
**Subtype**	435					<0.001
Classical		162 (37%)	122 (40%)	40 (31%)		
Mesenchymal		116 (27%)	99 (32%)	17 (13%)		
Proneural		157 (36%)	87 (28%)	70 (55%)		
**IDH-status**	966					<0.001
Mutant		531 (55%)	173 (36%)	358 (74%)		
Wildtype		435 (45%)	308 (64%)	127 (26%)		
**codel_1p19q**	940					<0.001
Codel		212 (23%)	27 (5.5%)	185 (41%)		
Non-codel		728 (77%)	462 (94%)	266 (59%)		
**Recurrence**	1,014					0.054
Primary		651 (64%)	314 (62%)	337 (66%)		
Recurrent		333 (33%)	171 (34%)	162 (32%)		
Secondary		30 (3.0%)	21 (4.2%)	9 (1.8%)		

^1^Mean (IQR); n (%).

^2^Welch Two Sample t-test; Pearson’s Chi-squared test.

**Figure 4:**
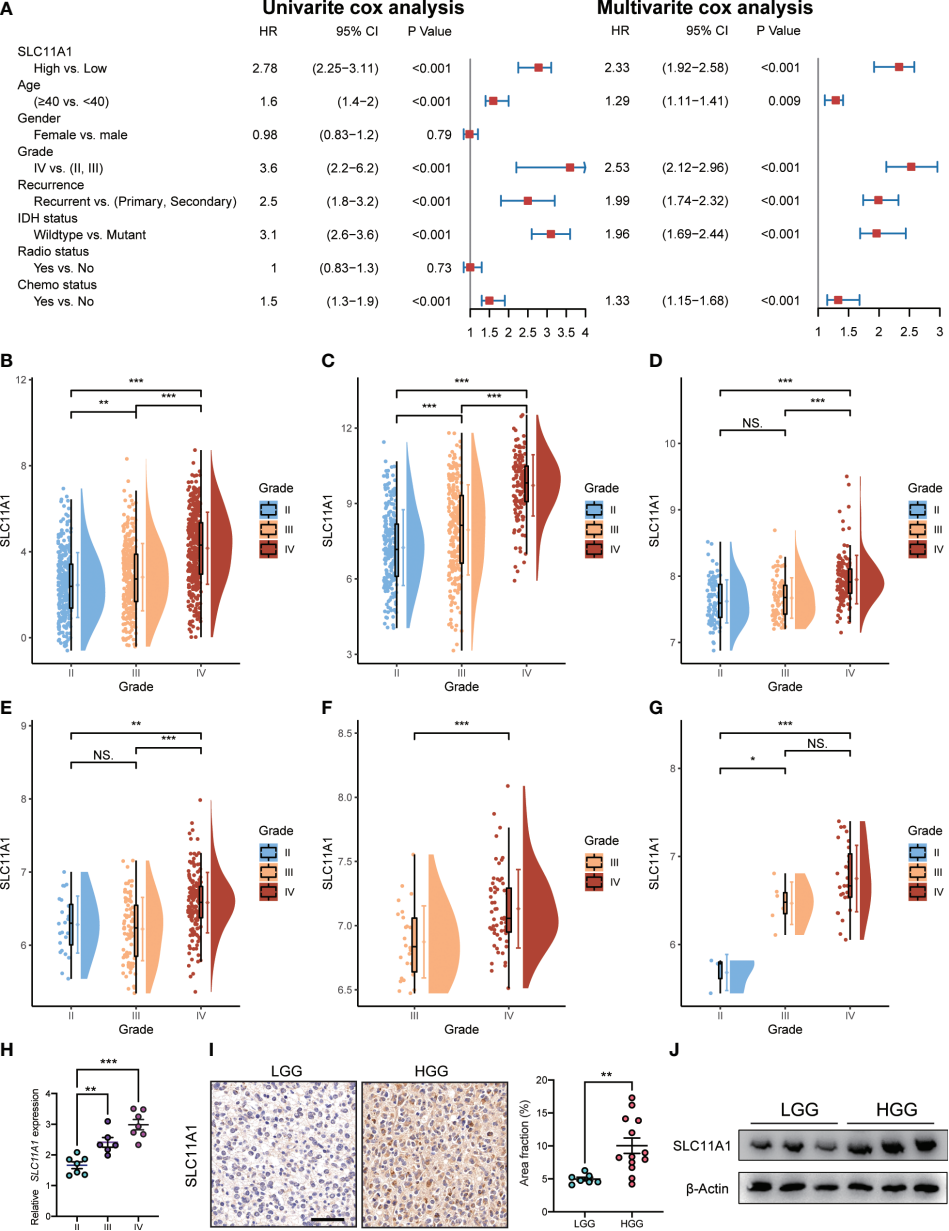
Expression of SLC11A1 increased with the progression of glioma. **(A)** Univariate and multivariate analysis for overall survival of glioma patients. **(B)** CGGA, **(C)** TCGA, **(D)** Rembrandt, **(E)** GSE16011, **(F)** GSE4412, and **(G)** GSE43289. (The X-axis represents the WHO grade while the Y-axis represents SLC11A1 expression value (log2). Based on Wilcoxon test.) **(H)** qRT-PCR of SLC11A1 of 20 patients with gliomas. **(I)** Representations and quantification of immunohistochemistry detection of SLC11A1 in LGG (low grade glioma) and HGG (high grade glioma). *,P < 0.05; **,P < 0.01; ***,P < 0.001, ns = no significance (Wilcoxon test). **(J)** Western blot of SLC11A1 in LGG (low grade glioma) and HGG (high grade glioma).

We used the Cox regression model to perform univariate and multivariate Cox analyses for 1018 glioma patients on different clinical variables. According to the results of the univariate Cox regression analysis (**Figure 3A**), SLC11A1 was an independent variable [high vs. low, HR=2.78, 95% CI (2.25-3.11)] for patients outcomes. Using the multivariate Cox model, SLC11A1 was also an independent determinant [high vs. low HR=2.33, 95% CI (1.92-2.58)] of the outcomes of glioma patients after controlling for grade, IDH status, age, chemotherapy status and recurrence (**Figure 4A**).

To determine the SLC11A1 expression in glioma patients with different tumor grades, we obtained data from a public database and our hospital, and we found that the expression of SLC11A1 was elevated in gliomas tissue with high malignant potential. In the CGGA dataset, the expression of SLC11A1 was notably higher in WHO grade III and IV tumors than in grade II tumors (**Figure 4B**). In TCGA dataset, an extremely increase in SLC11A1 expression was also noted in WHO grade IV and III tumors compared with grade II tumors (**Figure 4C**). In addition, a significant rising trend was observed in Rembrandt dataset: 98 patients with high SLC11A1 expression had grade II tumors, 85 had grade III tumors, and 130 had grade IV tumors (**Figure 4D**). Consistent with the above results, based on the analysis of GEO dataset analysis, the GSE16011 dataset showed an increasing trend in the number of patients with high-grade glioma (**Figure 4E**); similar results were observed for the GSE4412 dataset (**Figure 4F**) and the GSE43289 dataset (**Figure 4G**). qRT–PCR and immunohistochemistry (IHC) staining for SLC11A1 were used to evaluate SLC11A1 expression in tumor tissue samples and for further validation. Consistent with the above results, a dramatic increase in SLC11A1 was observed in HGG compared to LGG (**Figures 4H–J**). In conclusion, the SLC11A1 expression value was found to be a stable predictor of glioma patient survival. The expression value of SLC11A1 is a predictable predictor of prognosis for glioma patients.

### *SLC11A1* is correlated with immune activation and immune infiltration in gliomas

**Figure 5:**
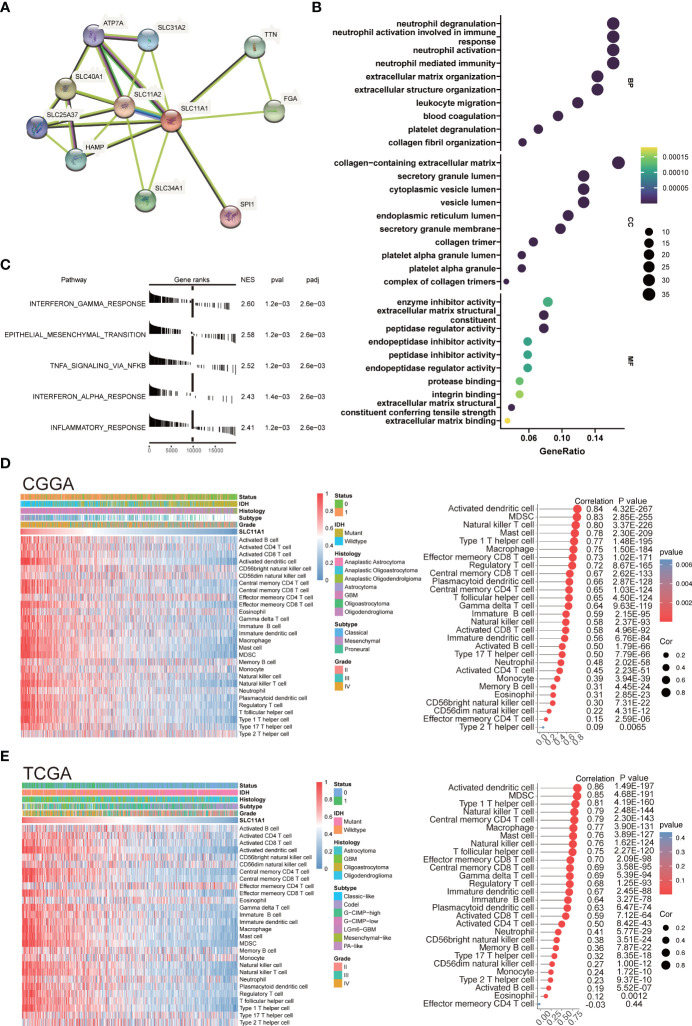
SLC11A1 is associated with immune infiltration and immune activation in gliomas. **(A)** STRING database shows the PPI network of SLC11A1. **(B)** GO (Gene Ontology) results for differential expression genes (Cut-off criteria for DEGs significance was adj. p value< 0.05 and the absolute value of the log2FC≥ 1.5). The X-axis represents gene ratio and the Y-axis represents different enriched pathways (BP: biological progress; CC: cellular component; MF: molecular function). **(C)** Rank-based gene set enrichment analysis shows significantly activated hallmark pathways in SLC11A1 high subgroup compared with low subgroup. **(D)** Heatmap showing SLC11A1-associated relative abundance of 28 immune cells in gliomas (CGGA), annotations show corresponding clinical features of each sample. **(E)** Heatmap showing SLC11A1-associated relative abundance of 28 immune cells in gliomas (TCGA), annotations show corresponding clinical features of each sample.

SLC11A1 (Nramp-1) is a strong candidate target for influencing autoimmune and infectious disease susceptibility (33). Researchers revealed that SLC11A1 regulates immune-inflammatory genes in macrophages when pristane induces arthritis in mice (34). An association was also found between functional SLC11A1 and enhanced generation of IFN-γ-producing T cells, which was related to phagosomal acidification and phagocytosis in dendritic cells (DCs) (35) (**Figure 5A**).

Therefore, our study was aimed at discovering how SLC11A1 expression relates to immune infiltration in gliomas and examining the molecular mechanisms by which SLC11A1 plays a role. We analyzed the DEGs between the different SLC11A1 groups (based on the CGGA dataset). The result indicates that 179 genes were upregulated, while 136 genes were downregulated. Then, the GO terms and KEGG pathways were annotated. In terms of GO biological processes, the enrichment expression of DEGs were focused on neutrophil activation involved in immune response, neutrophil degranulation, collagen-containing extracellular matrix, and leukocyte migration (**Figure 5B** and **Supplementary Figure 2B**). Following on from the KEGG results, complement and coagulation cascades, ECM-receptor interactions and Staphylococcus aureus infection were significantly enriched (**Supplementary Figure 2A** and **Supplementary Figure 2C**). Additionally, we explored the mechanisms underlying SLC11A1 in gliomas through gene set enrichment analysis (GSEA). These results indicated that various tumor progression- and immune activation-associated pathways, particularly extracellular matrix organization, cytokine signaling in the immune system and interferon alpha/beta signaling, activated inflammation and reflecting relatively enhanced tumor progression, were enriched in the high SLC11A1 subgroup (**Supplementary Figure 2D**). We also performed enrichment analysis by using the “HALLMARK” gene set, and consistent with the above results, the results showed that the high SLC11A1 subgroup showed significant upregulation in the immune-related pathways and EMT pathway (**Figure 5C**).

An immune phenotype was quantified using gene sets. Spearman’s test (**Supplementary Figure 3A**) indicated a high correlation between SLC11A1 and positive regulation of the inflammatory response. Along with increased expression of SLC11A1, the immune phenotype showed a “hot” tendency. In line with the above findings, SLC11A1 is a critical factor in the activation of the immune response in gliomas.

Next, it was observed that SLC11A1 levels correlate with immune infiltration, resulting in the discovery of possible mechanisms and roles involved in glioma, as well as its potential use for prognosis assessment. The relative abundance of 28 immune cell types in the CGGA cohort was systematically assessed using the ssGSEA algorithm. The association of SLC11A1 expression with infiltrating immune cells level was estimated by the Spearman method, which showed a tight knit connection between SLC11A1 and macrophages, B cells and T cells (**Figure 5D** and **Supplementary Figure 3B**). The TCGA cohort was treated as a validation set, and the results were highly consistent with the above results, which revealed that the SLC11A1 expression was associated with immune infiltration and microenvironment remodeling within gliomas (**Figure 5E** and **Supplementary Figure 3C**).

### *SLC11A1* indicates the TME phenotype in gliomas

**Figure 6:**
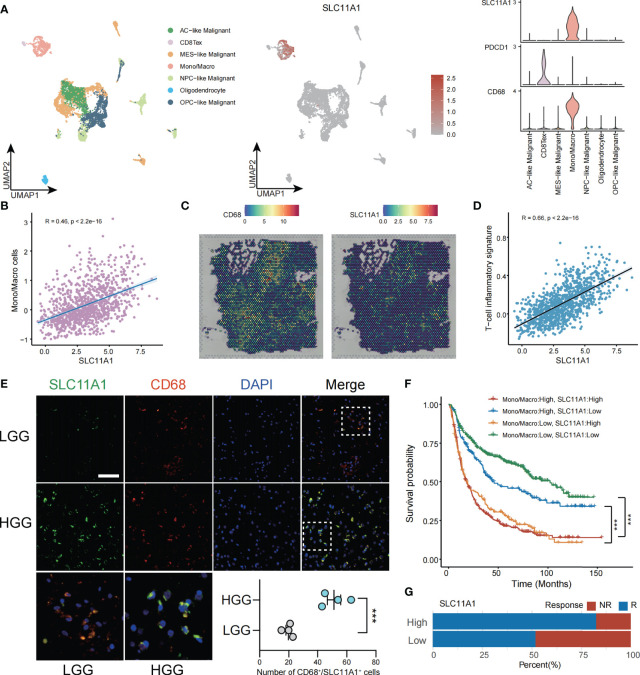
SLC11A1 expression implies TME in gliomas. **(A)** Analysis of immune cell infiltration and SLC11A1’s specific expression through single cell data of gliomas. **(B)** Correlation between the SLC11A1’s expression and Mono/Macro cells **(C)** Analysis of SLC11A1’s location in gliomas based on spatial transcriptome. **(D)** Correlation between the SLC11A1’s expression and T-cell inflammatory signature. **(E)** Representative immunofluorescence images of human glioma samples co-stained with PDCD1 or CD68 (red) and SLC11A1 (green) in LGG and HGG. **(F)** Kaplan-Meier plots were performed in context of monocytes/macrophages infiltration and SLC11A1 expression. ***,P < 0.001 (Wilcoxon test). **(G)** Rates of anti-PD1 responses of patients from the CGGA cohort in the high or low SLC11A1 subgroups based on ImmunCellAI.

To deepen our understanding of SLC11A1’s function in glioma’s tumor immune microenvironment (TIME), we utilized glioma single-cell data derived from the GEO dataset (GSE131928) (9), unsupervised analyses of this data identified 7 cell states representing the stromal, immune, and neoplastic cells commonly observed in glioma. According to the marker genes in the study of Neftel et al, neoplastic cells were split across four pan-glioma cell states, AC-like (EGFR), MES-like (CHI3L1), NPC-like (ELAVL4) and OPC-like (PDGFRA) (9), that observed across many glioma single-cell studies (36, 37). We also identified macrophages/monocytes by C1QB, C1QC and FCER1G (38), CD8 Tex cells by CD3D and IFITM1 (39), and oligodendrocyte by ERMN and KLK6 (40). The top 10 marker genes corresponding to these cell clusters were shown in the **Supplementary Figure 4A**. As shown in **Figure 6A**, SLC11A1 was specifically expressed in macrophages. We calculated the correlation between SLC11A1 and myeloid cells using the MCP-counter algorithm. The results also indicated that Macrophage infiltration and SLC11A1 expression were positively correlated. Moreover, we validated the specific expression of SLC11A1 on macrophages using spatial transcriptomic data (**Figures 6A–C**).

ESTIMATE is a tool (41) used gene expression level data to exhibit the existence of stromal/immune cells infiltrating tumor tissue. We used this method to calculate ESTIMATE scores for glioma patients, and the results showed that SLC11A1 was highly positively associated with stromal scores, immune scores and ESTIMATE scores but significantly negatively associated with glioma tumor purity (**Supplementary Figure 5A**). Then, we calculated TIS scores in CGGA patients. The results showed that the expression of SLC11A1 was positively associated with the TIS score (R=0.66, p < 0.001), indicating that high SLC11A1 expression may respond well to the anti-PD-L1 checkpoint inhibitor pembrolizumab (**Figure 6D**).

Immunofluorescence experiments demonstrated that the increase in SLC11A1 expression was accompanied by increased malignant potential and increased macrophage and T cell infiltration. Consistent with the above results, as shown in **Figure 6E**, SLC11A1 and CD68 colocalized in macrophages but not in CD8+ T cells. The expression of SLC11A1 could indicate the state of the TIME and could be an indicator of the response to immunotherapy (**Figure 6E**). When analyzed in the context of high or low infiltration of monocytes/macrophages (**Figure 6F**), low expression of SLC11A1 could predict increased survival independent of the expression of monocyte/macrophage markers. Patients with low expression level of SLC11A1 had a longer survival time when monocytes/macrophages were highly infiltrated, indicating that targeting SLC11A1 may lead to favorable treatment outcomes for patients with high levels of infiltrating monocytes/macrophages. In addition, we also predicted the response to immunotherapy in subgroups of gliomas with high and low expression of SLC11A1, and the results showed that the subgroup with high expression of SLC11A1 had a higher proportion of response to immunotherapy compared to the low expression group(**Figure 6G**).

**Figure 7:**
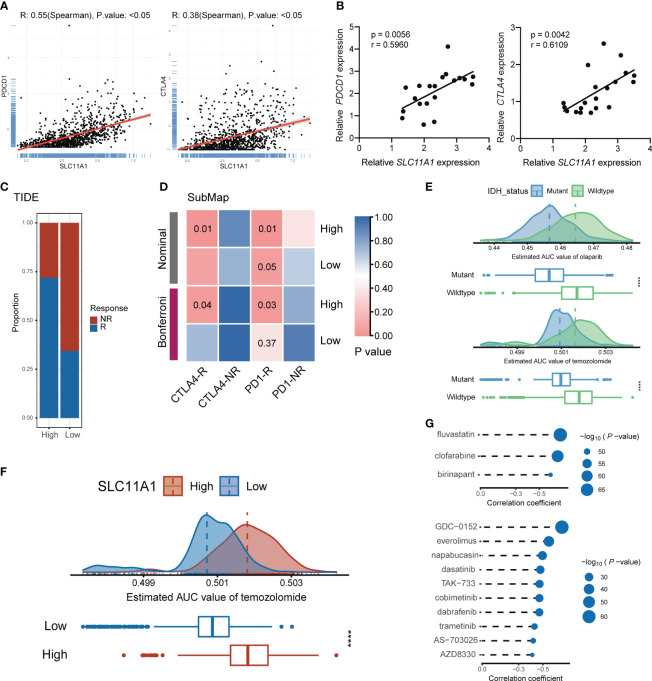
Subgroup divided by SLC11A1 predict potential immunotherapy responses of gliomas and identification of candidate agents with higher drug sensitivity in glioma patients. **(E)** Comparison of estimated olaparib (up) and temozolomide’s (down) sensitivity (logAUC) between IDH mutant and wildtype groups. **(F)** Comparison of estimated temozolomide’s sensitivity (logAUC) between high-SLC11A1 and low-SLC11A1 groups. **(G)** Spearman’s correlation analysis of three CTRP-derived compounds (up) and ten PRISM-derived compounds (down).

In the next step, the immune cells infiltration levels was calculated by using several independent algorithms with TIMER website (24) based on pancancer expression data. Consistent with previous research, SLC11A1 was positively associated with the levels of many types of infiltrating immune cells (**Supplementary Figure 5B**). Notably, SLC11A1 was positively associated with the infiltration of macrophages and monocytes. As tumor progression and immune activation were both enhanced in the high SLC11A1 group, SLC11A1 expression possibly be related to the high PD-1 and CTLA4 expression. Analysis of linear regression showed a significant relationship between SLC11A1 and PDCD1 and CTLA4 (**Figure 7A**). A similar conclusion was drawn from the TCGA dataset (**Supplementary Figure 6A**). High SLC11A1 subgroups exhibited both immunologic activation and immunologic suppression. The phenomenon exhibits the phenomenon that immune activation was enriched in the high SLC11A1 subgroup while tumor progression was not suppressed.

### *SLC11A1* expression level predicts the immunotherapy response of glioma patients

To identify the transcriptome results from public datasets, 20 patients were included from Shanghai General Hospital, and the association between the expression levels of PDCD1, CTLA4 and SLC11A1 by quantitative real-time PCR were investigated. The results indicated that SLC11A1 was positively associated with PDCD1 and CTLA4 (**Figure 7B**). SLC11A1-expressing patients were found to have high expression of the therapeutic targets CTLA4 and PD-1/PD-L1, which indicated that ICB treatment may be effective.

To evaluate the effect of SLC11A1 in immunotherapy response, we utilized some tools. ImmunCellAI (26) suggested that glioma patients with a high expression levels of SLC11A1 are more inclined to respond to immunotherapy (79%, 406/509) than patients with low SLC11A1 levels (53%, 273/509) (**Figure 6G**), and TIDE (27) revealed a similar conclusion (High: 72%, 366/509; Low: 35%, 178/509) (**Figure 7C**). In order to make a comparison about the similarity of the expression profiles between previous melanoma patients with detailed immunotherapeutic information and the SLC11A1 subgroups of glioma patients, we also utilized the submap algorithm, which demonstrated that the patients in the SLC11A1-high subgroup were more reactive to anti-PD-1 and anti-CTLA4 treatment (**Figure 7D**) (28). These results were consistent with previous findings.

In conclusion, the SLC11A1 gene may be a useful indicator of the phenotype of the immune microenvironment within a tumor and may help to predict immunotherapy response in patients with gliomas.

### Estimation of drug response and identification of potential therapeutic agents for glioma patients with high or low *SLC11A1* expression

The PRISM and CTRP datasets include drug sensitivity profiles and gene expression profiles of hundreds of CCLs that can be used to develop a drug response prediction model. With the *pRRophetic* package, we can predict the drug-sensitive patients, and further obtain the AUC valuation of each compound in clinical sample.

Before further analysis, we first showed that estimation of drug response is an accurate and reliable method, and we followed a similar process as described in a previous study (42). Temozolomide (a first-line chemotherapeutic drug used in the treatment of glioblastoma multiforme and anaplastic astrocytoma) and olaparib (a PARP inhibitor) were employed to determine whether the estimated immunotherapeutic response matched the actual clinical response. Recent studies revealed that IDH-mutant gliomas could be vulnerable to PARP inhibitor and temozolomide treatment. Therefore, we divided patients from the CGGA cohort into two different groups based on the IDH alteration status. The difference in the AUC valuation of olaparib and temozolomide between the two groups was compared by the Wilcoxon rank-sum test, while the results showed that the estimated AUC values were extremely lower in patients with mutations in IDH (*p* < 0.001, **Figure 7E**). The results illustrated that patients with IDH mutations were more sensitive to chemotherapy drugs, which is consistent with the actual clinical response to olaparib and temozolomide.

Similarly, glioma patients were classified into two groups (high vs. low SLC11A1 expression group) based on the median SLC11A1 level. We utilized the same methodology to assess the sensitivity of different SLC11A1 subgroups to temozolomide. We demonstrated that patients with low expression of SLC11A1 showed significantly lower estimated AUC values of temozolomide (p < 0.0001, **Figure 7F**). This result suggested that the low SLC11A1 subgroup was more sensitive to temozolomide. Although temozolomide is the first-line drug for glioma treatment, because of the heterogeneity of glioma, patients are prone to develop drug resistance, so there is an urgent clinical need for novel drugs targeting new molecules. A variety of drug candidates with higher efficacy are required in clinical treatment.

CTRP and PRISM can be used to find drug candidates. Therefore, response analysis of differential drug between the high-SLC11A1 subgroup and the low-SLC11A1 subgroup was performed to identify compounds with low estimated AUC values (log_2_FC > 0.10). The Spearman correlation coefficient between expression of SLC11A1 and AUC value was tested to select compounds that are negatively correlated. Based on these analyses, we identified three CTRP-derived compounds (fluvastatin, clofarabine and birinapant) and ten PRISM-derived compounds (GDC-0152, everolimus, napabucasin, dasatinib, TAK-733, cobimetinib, dabrafenib, trametinib, AS-703026 and AZD8330). SLC11A1 was negatively correlated with all of these compounds, and the estimated AUC values were lower in the high-SLC11A1 group (p < 0.001); the most notable candidates included clofarabine (mean (IQR), low: 0.3850 (0.3694, 0.4017) vs. high: 0.3547 (0.3377, 0.3668)) in the CTRP-derived compound group and AZD8330 [mean (IQR), low: 0.1058 (0.0975, 0.1125) vs. high: 0.0960 (0.0884, 0.1038)] in the PRISM-derived compound group (**Figure 7G** and **Supplementary Figure 7A**). In previous study (43), fluvastatin inhibits the growth and alters the malignant phenotype of the glioma cell line. The inhibitory effects of fluvastatin on cell proliferation is associated with decreased p-ERK1/2 expression, upregulation of p-JNK1/2. Fluvastatin has high anticancer activity and lacks toxicity to normal cells, suggesting the potential use of this statin for the treatment of gliomas (43). Clofarabine is a purine nucleoside analog drug used in the treatment of hematological malignancies (44). Birinapant has extensive IAP antagonistic effects. Birinapant can cause rapid degradation of cIAP1, cleavage of PARP, activation of caspase, and inhibition of activation of NF-κB (45). The use of clofarabine and birinapant in gliomas has not been reported. AZD8330 inhibits growth factor-mediated cell signaling and tumor cell proliferation by inhibiting MEK1/2. Yi’s study showed that YAP/TAZ depletion with MEK inhibition results in a durable suppression of NF2 tumors (46), indicating MEK inhibitor like AZD8330 could be used in many tumors.

In conclusion, it is reasonable to assume that the level of SLC11A1 expression can also be used as an indicator to evaluate the sensitivity to temozolomide. Moreover, in our present study, new target drugs that may address the current situation of temozolomide resistance in the treatment of glioma were screened based on SLC11A1 expression, and clofarabine and AZD8830 may be potential options for further basic research and clinical strategy development in the future.

## Discussion

Because gliomas are highly heterogeneous, each patient’s course and therapeutic effect may be unique (47). Thus, to manage gliomas in a comprehensive manner, the patient’s individual characteristics, clinical symptoms, and tumor progression need to be considered (48). Currently, genetic examination is widely used for the precise diagnosis and evaluation of therapeutic effects. For example, it has been illustrated that patients with IDH1 and IDH2 mutations tend to have favorable outcomes and are also more susceptible to radiotherapy and chemotherapy (49, 50). In addition, patients with the 1p19q codeletion are considered ineligible for radiotherapy (51, 52). Anti-SLC11A1 immunotherapy is a suitable treatment option for glioma, as shown in this study.

Generally, the appreciation for the TME as a determining factor of cancer outcome is growing. In the process of tumorigenesis, a protumor TME is formed that includes stromal cells, fibroblasts, macrophages, as well as vascular endothelial cells and their secretory chemokines. These cells interact tightly and dynamically, and the balance of cytokine production and metabolite production changes over time (53). At the beginning of the process, tumor cells can attract and activate infiltrating immune cells to exert antitumor functions and impede tumor progression. However, in the late period, immune cells can play both antitumor and protumor roles. When this balance is disrupted, immune evasion and tumor progression are further promoted (54, 55). As a hot topic in tumor research, recent decades have seen remarkable advances in tumor immunotherapy research (56). ICB treatment can block inhibitory signaling, directly activate cytotoxic T lymphocytes to achieve antitumor effects, and may serve as an effective therapeutic strategy in patients with solid tumors (57). Despite the immune system’s ability to detect malignant tumor cells, the tumor microenvironment upregulates suppressive immune checkpoints, leading to weak anti-cancer immunity (58).

SLC11A1 is a phagosomal membrane protein located in monocytes (59), that serves as a proinflammatory factor and is closely correlated with the occurrence and progression of various inflammatory diseases (60). Besides, it is also related to susceptibility to infectious disease (33). SLC11A1 modulates immune activation (34). Despite this, few studies have evaluated whether SLC11A1 contributes to tumor progression. According to our current study, SLC11A1 is overexpressed in several kinds of cancer, and especially in gliomas. To further investigate the relationship between SLC11A1 and glioma, the cohort was split into two different groups based on SLC11A1 levels. The high SLC11A1 expression group greater malignant potential and a poorer clinical prognosis when compared with the low SLC11A1 expression group. Additionally, SLC11A1 expression was correlated to age, IDH mutation status, tumor malignancy, 1p/19q codeletion and higher TMB. TMB is taken as a key driver in immunogenic neopeptides generation, which are displayed on MHC molecules on the tumor cell surface, and regulates the patient’s response to immune checkpoint inhibitors (ICIs). According to the above results, SLC11A1 could be considered a potential practical predictor for prognosis evaluation and clinical diagnosis in glioma patients. We also observed that various immune activators and tumor progression-associated genes were enriched in high SLC11A1 groups, especially those related to cytokine signal transduction and PD-1 signal transduction. The cytokine signaling pathway and PD-1 signaling pathway are critical regulatory pathways in glioma immunotherapy (61). Therefore, we hypothesized that SLC11A1 may serve as a potential target for glioma treatment. This is the first study to prove that the SLC11A1 gene is a novel therapeutic and diagnostic target. In addition, we revealed the role of SLC11A1 in the development of glioma and assessed the underlying mechanism in immunotherapeutic response. In our present study, we revealed the relationship between SLC11A1 and the immunotherapeutic response, providing a potential therapeutic target for clinical diagnosis and management.

Glioma is highly heterogeneous, so it is almost impossible to explore a strategy suitable for all glioma cases. There is lacking corresponding biomarkers in all current therapies for glioma and thus satisfactory clinical effects cannot be achieved. Hence, finding individualized treatment strategies for glioma patients is important to maximize the therapeutic effects. We divided patients into two groups according to IDH mutation status. The differences of olaparib and temozolomide between the two groups were compared, and the results suggested that patients with mutation of IDH presented significantly lower estimated AUC values for both drugs, consistent with how the chemotherapeutic drugs behave clinically. Moreover, the patients with high SLC11A1 expression showed higher estimated AUC values for temozolomide, which indicates that glioma

Taken together, our study illustrates that SLC11A1 can serve as a novel indicator for clinical diagnosis, prognostic prediction, and immunotherapeutic response evaluation in glioma patients. The suggestion that SLC11A1 can be a practical immunotherapeutic target in glioma patients is reasonable. Furthermore, exploration of novel potential drugs, such as AZD8330 and clofarabine, may present a more robust and comprehensive perspective regarding their utilization. The above results are of great significance in clinical management and will be conducive to precise treatment and prognosis evaluation.

Studies have shown that the regulation of macrophage iron metabolism by SLC11A1 plays an important role in early macrophage activation, and previous studies have also shown that SLC11A1 is expressed only in phagocytes [i.e., monocytes/macrophages and granulocytes (PMNs)], which we also demonstrated in the present study, so to some extent SLC11A1 can reflect the number of macrophages in glioma. Moreover, increased macrophage infiltration suggests a suppressive immune microenvironment, which usually implies a poorer prognosis. We hypothesize that high expression of SLC11A1 could reflect a tumor microenvironment tending to be “cold”, which also provides an explanation for the association of high SLC11A1 expression with poor prognosis. Additionally, we observed that glioma patients with high expression of SLC11A1 were more sensitive to immunotherapy, while glioma patients with low expression of SLC11A1 responded better to temozolomide. Therefore, SLC11A1 may serve as an indicting factor for whether patients be treated with different treatment approaches (chemotherapy or immunotherapy). We yielded three CTRP-derived compounds (fluvastatin, clofarabine, and birinapant) and ten PRISM-derived compounds (GDC-0152, everolimus, napabucasin, dasatinib, TAK-733, cobimetinib, dabrafenib, trametinib, AS-703026, and AZD8330). In previous study, fluvastatin inhibits the growth and alters the malignant phenotype of the glioma cell line, suggesting the potential use of this statin for the treatment of gliomas (7). Furthermore, fluvastatin could suppress mitochondrial respiration through the synthesis inhibition of coenzyme Q and normalized T-cell-relative immune microenvironment, thereby effectively sensitizing the potency of Anti-PD1 against colorectal cancer in the MC38 homograft mouse model (13). Interestingly, a previous study reported that birinapant upregulates MHC-I, sensitizes cancer cells to T cell-dependent killing, and increases ICB efficacy (14). We wondered whether these two drugs could also play similar role in activating the immune system in glioma and achieving a sensitizing immunotherapeutic effect. Clofarabine is the drug granted approval for treatment of pediatric acute leukemia. Recent clinical studies have established the efficacy of clofarabine in treating malignancies with a poor prognosis (15). All these compounds, especially clofarabine and AZD8330 indicated the negative correlation of AUC value with SLC11A1 expression level. Although these two drugs have been reported to function in various cancers, no existing study had investigated their potential role and underlying mechanism in glioma management. There is an urgent need for further validation to explore novel clinical strategies.

## Data availability statement

The original contributions presented in the study are included in the article/**Supplementary Material**. Further inquiries can be directed to the corresponding authors.

## Ethics statement

The studies involving human participants were reviewed and approved by Shanghai General Hospital’s Human Investigation Ethical Committee. The patients/participants provided their written informed consent to participate in this study.

## Author contributions

HX, AZ, CF, and XW were involved in data analysis and interpretation. QZ, YL, and ZZ collected clinical data and tumor samples, and performed experiments. WW supported clinical data analysis. YX and ML designed the experiment, interpreted the data, and wrote the manuscript. All authors reviewed and approved the manuscript.

## Funding

This research received grants from 1) National Key R&D Program of China (2018YFA0108603); 2) Beijing Tianjin Hebei basic research cooperation project (19JCZDJC64600(Z)); 3) CAMS Innovation Fund for medical sciences(CIFMS) (2020-I2M-C&T-B-028); 4) Non-profit Central Research Institute Fund of Chinese Academy of Medical Sciences(2020-JKCS-026); 5) Cross Research Fund of Medicine and Engineering of Shanghai Jiao Tong University (YG2019QNA67).

## Acknowledgments

The authors would like to thank Qiyu Gong from Shanghai Jiao Tong University for her generous and outstanding help in this research design. We thank openbiox community and Hiplot team (https://hiplot.com.cn) for providing technical assistance and valuable tools for data analysis and visualization.

## Conflict of interest

The authors declare that the research was conducted in the absence of any commercial or financial relationships that could be construed as a potential conflict of interest.

## Publisher’s note

All claims expressed in this article are solely those of the authors and do not necessarily represent those of their affiliated organizations, or those of the publisher, the editors and the reviewers. Any product that may be evaluated in this article, or claim that may be made by its manufacturer, is not guaranteed or endorsed by the publisher.

## References

[1] Huang K Fang C Yi K Liu X Qi H Tan Y. The role of PTRF/Cavin1 as a biomarker in both glioma and serum exosomes. Theranostics (2018) 8(6):1540–57. doi: 10.7150/thno.22952 PMC5858166 29556340

[2] Huang W Zhong Z Luo C Xiao Y Li L Zhang X. The miR-26a/AP-2α/Nanog signaling axis mediates stem cell self-renewal and temozolomide resistance in glioma. Theranostics (2019) 9(19):5497–516. doi: 10.7150/thno.33800 PMC6735392 31534499

[3] Gao YF Liu JY Mao XY He ZW Zhu T Wang ZB. LncRNA FOXD1-AS1 acts as a potential oncogenic biomarker in glioma. CNS Neurosci Ther (2020) 26(1):66–75. doi: 10.1111/cns.13152 31102349 PMC6930828

[4] Cunrath O Bumann D. Host resistance factor SLC11A1 restricts salmonella growth through magnesium deprivation. Science (2019) 366(6468):995–9. doi: 10.1126/science.aax7898 31753999

[5] Decobert M Larue H Bergeron A Harel F Pfister C Rousseau F. Polymorphisms of the human NRAMP1 gene are associated with response to bacillus calmette-guerin immunotherapy for superficial bladder cancer. J Urol (2006) 175(4):1506–11. doi: 10.1016/S0022-5347(05)00653-1 16516037

[6] Zaahl MG Warnich L Victor TC Kotze MJ. Association of functional polymorphisms of SLC11A1 with risk of esophageal cancer in the south African colored population. Cancer Genet Cytogenet (2005) 159(1):48–52. doi: 10.1016/j.cancergencyto.2004.09.017 15860357

[7] Wang S. (2020).

[8] Bowman RL Wang Q Carro A Verhaak RG Squatrito M. GlioVis data portal for visualization and analysis of brain tumor expression datasets. Neuro Oncol (2017) 19(1):139–41. doi: 10.1093/neuonc/now247 PMC5193031 28031383

[9] Neftel C Laffy J Filbin MG Hara T Shore ME Rahme GJ. An integrative model of cellular states, plasticity, and genetics for glioblastoma. Cell (2019) 178(4):835–849.e21. doi: 10.1016/j.cell.2019.06.024 31327527 PMC6703186

[10] Hao YH Hao S Andersen-Nissen E Mauck WM Zheng SW Butler A. Integrated analysis of multimodal single-cell data. Cell (2021) 184(13):3573–3587.e29. doi: 10.1016/j.cell.2021.04.048 34062119 PMC8238499

[11] Aran D Looney AP Liu LQ Wu E Fong V Hsu A. Reference-based analysis of lung single-cell sequencing reveals a transitional profibrotic macrophage. Nat Immunol (2019) 20(2):163–72. doi: 10.1038/s41590-018-0276-y PMC6340744 30643263

[12] Sun DQ Wang J Han Y Dong X Ge J Zheng RB. TISCH: a comprehensive web resource enabling interactive single-cell transcriptome visualization of tumor microenvironment. Nucleic Acids Res (2021) 49(D1):D1420–30. doi: 10.1093/nar/gkaa1020 PMC7778907 33179754

[13] Ellrott K Bailey MH Saksena G Covington KR Kandoth C Stewart C. Scalable open science approach for mutation calling of tumor exomes using multiple genomic pipelines. Cell Syst (2018) 6(3):271–281.e7. doi: 10.1016/j.cels.2018.03.002 29596782 PMC6075717

[14] Mayakonda A Lin DC Assenov Y Plass C Koeffler HP. Maftools: efficient and comprehensive analysis of somatic variants in cancer. Genome Res (2018) 28(11):1747–56. doi: 10.1101/gr.239244.118 PMC6211645 30341162

[15] Ritchie ME Phipson B Wu D Hu YF Law CW Shi W. Limma powers differential expression analyses for RNA-sequencing and microarray studies. Nucleic Acids Res (2015) 43(7):e47. doi: 10.1093/nar/gkv007 25605792 PMC4402510

[16] Yu G Wang LG Han Y He QY. clusterProfiler: an r package for comparing biological themes among gene clusters. Omics (2012) 16(5):284–7. doi: 10.1089/omi.2011.0118 PMC3339379 22455463

[17] Fang H De Wolf H Knezevic B Burnham KL Osgood J Sanniti A. A genetics-led approach defines the drug target landscape of 30 immune-related traits. Nat Genet (2019) 51(7):1082–91. doi: 10.1038/s41588-019-0456-1 PMC7124888 31253980

[18] García-Mulero S Alonso MH Pardo J Santos C Sanjuan X Salazar R. Lung metastases share common immune features regardless of primary tumor origin. J Immunother Cancer (2020) 8(1):e000491. doi: 10.1136/jitc-2019-000491 32591432 PMC7319789

[19] Hänzelmann S Castelo R Guinney J. GSVA: gene set variation analysis for microarray and RNA-seq data. BMC Bioinf (2013) 14:7. doi: 10.1186/1471-2105-14-7 PMC3618321 23323831

[20] Jia Q Wu W Wang Y Alexander PB Sun C Gong Z. Local mutational diversity drives intratumoral immune heterogeneity in non-small cell lung cancer. Nat Commun (2018) 9(1):5361. doi: 10.1038/s41467-018-07767-w 30560866 PMC6299138

[21] Newman AM Liu CL Green MR Gentles AJ Feng WG Xu Y. Robust enumeration of cell subsets from tissue expression profiles. Nature Methods (2015) 12(5):453–7. doi: 10.1038/nmeth.3337 PMC4739640 25822800

[22] Becht E Giraldo NA Lacroix L Buttard B Elarouci N Petitprez F. Estimating the population abundance of tissue-infiltrating immune and stromal cell populations using gene expression. Genome Biol (2016) 17(1):218. doi: 10.1186/s13059-016-1070-5 27765066 PMC5073889

[23] Finotello F Mayer C Plattner C Laschober G Rieder D Hackl H. Molecular and pharmacological modulators of the tumor immune contexture revealed by deconvolution of RNA-seq data. Genome Med (2019) 11(1):34. doi: 10.1186/s13073-019-0638-6 31126321 PMC6534875

[24] Li TW Fu JX Zeng ZX Cohen D Li J Chen QM. TIMER2.0 for analysis of tumor-infiltrating immune cells. Nucleic Acids Res (2020) 48(W1):W509–14. doi: 10.1093/nar/gkaa407 PMC7319575 32442275

[25] Aran D Hu ZC Butte AJ. xCell: digitally portraying the tissue cellular heterogeneity landscape. Genome Biol (2017) 18(1):220. doi: 10.1186/s13059-017-1349-1 29141660 PMC5688663

[26] Miao YR Zhang Q Lei Q Luo M Xie GY Wang H. ImmuCellAI: A unique method for comprehensive T-cell subsets abundance prediction and its application in cancer immunotherapy. Adv Sci (Weinh) (2020) 7(7):1902880. doi: 10.1002/advs.201902880 32274301 PMC7141005

[27] Jiang P Gu S Pan D Fu J Sahu A Hu X. Signatures of T cell dysfunction and exclusion predict cancer immunotherapy response. Nat Med (2018) 24(10):1550–8. doi: 10.1038/s41591-018-0136-1 PMC6487502 30127393

[28] Hoshida Y Brunet JP Tamayo P Golub TR Mesirov JP. Subclass mapping: identifying common subtypes in independent disease data sets. PLos One (2007) 2(11):e1195. doi: 10.1371/journal.pone.0001195 18030330 PMC2065909

[29] Geeleher P Cox N Huang RS. pRRophetic: An r package for prediction of clinical chemotherapeutic response from tumor gene expression levels. PLos One (2014) 9(9):e107468. doi: 10.1371/journal.pone.0107468 25229481 PMC4167990

[30] Balduzzi S Rucker G Schwarzer G. How to perform a meta-analysis with r: a practical tutorial. Evidence-Based Ment Health (2019) 22(4):153–60. doi: 10.1136/ebmental-2019-300117 PMC10231495 31563865

[31] Gu ZG Eils R Schlesner M. Complex heatmaps reveal patterns and correlations in multidimensional genomic data. Bioinformatics (2016) 32(18):2847–9. doi: 10.1093/bioinformatics/btw313 27207943

[32] Miller CA McMichael J Dang HX Maher CA Ding L Ley TJ. Visualizing tumor evolution with the fishplot package for r. BMC Genomics (2016) 17(1):880. doi: 10.1186/s12864-016-3195-z 27821060 PMC5100182

[33] Braliou GG Kontou PI Boleti H Bagos PG. Susceptibility to leishmaniasis is affected by host SLC11A1 gene polymorphisms: a systematic review and meta-analysis. Parasitol Res (2019) 118(8):2329–42. doi: 10.1007/s00436-019-06374-y 31230160

[34] Correa MA Canhamero T Borrego A Katz ISS Jensen JR Guerra JL. Slc11a1 (Nramp-1) gene modulates immune-inflammation genes in macrophages during pristane-induced arthritis in mice. Inflammation Res (2017) 66(11):969–80. doi: 10.1007/s00011-017-1077-8 28669029

[35] Dai YD Marrero IG Gros P Zaghouani H Wicker LS Sercarz EE. Slc11a1 enhances the autoimmune diabetogenic T-cell response by altering processing and presentation of pancreatic islet antigens. Diabetes (2009) 58(1):156–64. doi: 10.2337/db07-1608 PMC2606865 18984740

[36] Coy S Wang S Stopka SA Lin JR Yapp C Ritch CC. Single cell spatial analysis reveals the topology of immunomodulatory purinergic signaling in glioblastoma. Nat Commun (2022) 13(1):4814. doi: 10.1038/s41467-022-32430-w 35973991 PMC9381513

[37] Abdelfattah N Kumar P Wang CY Leu JS Flynn WF Gao RL. Single-cell analysis of human glioma and immune cells identifies S100A4 as an immunotherapy target. Nat Commun (2022) 13(1):767. doi: 10.1038/s41467-022-28372-y 35140215 PMC8828877

[38] Dong KQ Chen WJ Pan XW Wang HR Sun Y Qian C. FCER1G positively relates to macrophage infiltration in clear cell renal cell carcinoma and contributes to unfavorable prognosis by regulating tumor immunity. BMC Cancer (2022) 22(1):140. doi: 10.1186/s12885-022-09251-7 35120484 PMC8815209

[39] He QF Xu Y Li J Huang ZM Li XH Wang XC. CD8(+) T-cell exhaustion in cancer: mechanisms and new area for cancer immunotherapy. Briefings Funct Genomics (2019) 18(2):99–106. doi: 10.1093/bfgp/ely006 29554204

[40] Murakami K Jiang YP Tanaka T Bando Y Mitrovic B Yoshida S. In vivo analysis of kallikrein-related peptidase 6 (KLK6) function in oligodendrocyte development and the expression of myelin proteins. Neuroscience (2013) 236:1–11. doi: 10.1016/j.neuroscience 23376368

[41] Yoshihara K Shahmoradgoli M Martinez E Vegesna R Kim H Torres-Garcia W. Inferring tumour purity and stromal and immune cell admixture from expression data. Nat Commun (2013) 4:2612. doi: 10.1038/ncomms3612 24113773 PMC3826632

[42] Yang C Huang X Li Y Chen J Lv Y Dai S. Prognosis and personalized treatment prediction in TP53-mutant hepatocellular carcinoma: an in silico strategy towards precision oncology. Briefings Bioinf (2021) 22(3):bbaa164. doi: 10.1093/bib/bbaa164 32789496

[43] Slawinska-Brych A Zdzisinska B Kandefer-Szerszen M. Fluvastatin inhibits growth and alters the malignant phenotype of the C6 glioma cell line. Pharmacol Rep (2014) 66(1):121–9. doi: 10.1016/j.pharep.2014.01.002 24905317

[44] Lee YJ Lee YJ Im JH Won SY Kim YB Cho MK. Synergistic anti-cancer effects of resveratrol and chemotherapeutic agent clofarabine against human malignant mesothelioma MSTO-211H cells. Food Chem Toxicol (2013) 52:61–8. doi: 10.1016/j.fct.2012.10.060 23146690

[45] Singh T Neal A Dibernardo G Raheseparian N Moatamed NA Memarzadeh S. Efficacy of birinapant in combination with carboplatin in targeting platinum-resistant epithelial ovarian cancers. Int J Oncol (2022) 60(3):35. doi: 10.3892/ijo.2022.5325 35191515 PMC8878637

[46] White SM Avantaggiati ML Nemazanyy I Di Poto C Yang Y Pende M. YAP/TAZ inhibition induces metabolic and signaling rewiring resulting in targetable vulnerabilities in NF2-deficient tumor cells. Dev Cell (2019) 49(3):425–443.e9. doi: 10.1016/j.devcel.2019.04.014 31063758 PMC6524954

[47] Wan RJ Peng W Xia QX Zhou HH Mao XY. Ferroptosis-related gene signature predicts prognosis and immunotherapy in glioma. CNS Neurosci Ther (2021) 27(8):973–986. doi: 10.1111/cns.13654 PMC8265949 33969928

[48] Nicholson JG Fine HA. Diffuse glioma heterogeneity and its therapeutic implications. Cancer Discovery (2021) 11(3):575–90. doi: 10.1158/2159-8290.CD-20-1474 33558264

[49] Di L Wang CP Shah AH Eichberg DG Semonche AM Sanjurjo AD. A cohort study on prognostic factors for laser interstitial thermal therapy success in newly diagnosed glioblastoma. Neurosurgery (2021) 89(3):496–503. doi: 10.1093/neuros/nyab193 PMC8364818 34156076

[50] Xiong Z Yang Q Li X. Effect of intra- and inter-tumoral heterogeneity on molecular characteristics of primary IDH-wild type glioblastoma revealed by single-cell analysis. CNS Neurosci Ther (2020) 26(9):981–9. doi: 10.1111/cns.13396 PMC7415209 32488994

[51] McNamara MG Jiang H Lim-Fat MJ Sahebjam S Kiehl TR Karamchandani J. Treatment outcomes in 1p19q Co-deleted/Partially deleted gliomas. Can J Neurol Sci (2017) 44(3):288–94. doi: 10.1017/cjn.2016.420 28488951

[52] Wang J Yan L Ai P He Y Guan H Wei Z. Observation versus radiotherapy with or without temozolomide in postoperative WHO grade II high-risk low-grade glioma: a retrospective cohort study. Neurosurg Rev (2021) 44(3):1447–55. doi: 10.1007/s10143-020-01326-y 32529528

[53] Economopoulou P Kotsantis I Psyrri A. Tumor microenvironment and immunotherapy response in head and neck cancer. Cancers (Basel) (2020) 12(11):3377. doi: 10.3390/cancers12113377 33203092 PMC7696050

[54] Gajewski TF Schreiber H Fu YX. Innate and adaptive immune cells in the tumor microenvironment. Nat Immunol (2013) 14(10):1014–22. doi: 10.1038/ni.2703 PMC4118725 24048123

[55] Sistigu A Musella M Galassi C Vitale I De Maria R. Tuning cancer fate: Tumor microenvironment's role in cancer stem cell quiescence and reawakening. Front Immunol (2020) 11:2166. doi: 10.3389/fimmu.2020.02166 33193295 PMC7609361

[56] He Y Chen L Zhao L Dang S Liu G Sasada S. Genomic and transcriptional alterations in first-line chemotherapy exert a potentially unfavorable influence on subsequent immunotherapy in NSCLC. Theranostics (2021) 11(14):7092–109. doi: 10.7150/thno.58039 PMC8171101 34093873

[57] Kuzume A Chi S Yamauchi N Minami Y. Immune-checkpoint blockade therapy in lymphoma. Int J Mol Sci (2020) 21(15):5456. doi: 10.3390/ijms21155456 32751706 PMC7432396

[58] Aslan K Turco V Blobner J Sonner JK Liuzzi AR Núñez NG. Heterogeneity of response to immune checkpoint blockade in hypermutated experimental gliomas. Nat Commun (2020) 11(1):931. doi: 10.1038/s41467-020-14642-0 32071302 PMC7028933

[59] Bauler TJ Starr T Nagy TA Sridhar S Scott D Winkler CW. Salmonella meningitis associated with monocyte infiltration in mice. Am J Pathol (2017) 187(1):187–99. doi: 10.1016/j.ajpath.2016.09.002 PMC5225301 27955815

[60] Xue Y Li J Lu X. A novel immune-related prognostic signature for thyroid carcinoma. Technol Cancer Res Treat (2020) 19:1533033820935860. doi: 10.1177/1533033820935860 32588760 PMC7325536

[61] Tong L Li J Li Q Wang X Medikonda R Zhao T. ACT001 reduces the expression of PD-L1 by inhibiting the phosphorylation of STAT3 in glioblastoma. Theranostics (2020) 10(13):5943–56. doi: 10.7150/thno.41498 PMC7254983 32483429

