# Supplementary material for: SLC11A1 as a stratification indicator for immunotherapy or chemotherapy in patients with glioma
Source: Front Immunol. 2022 Nov 30;13:980378. doi: 10.3389/fimmu.2022.980378 (PMC9748290; doi:10.3389/fimmu.2022.980378)
Supplement: Supplementary file 2 [file DataSheet_1.docx]

**
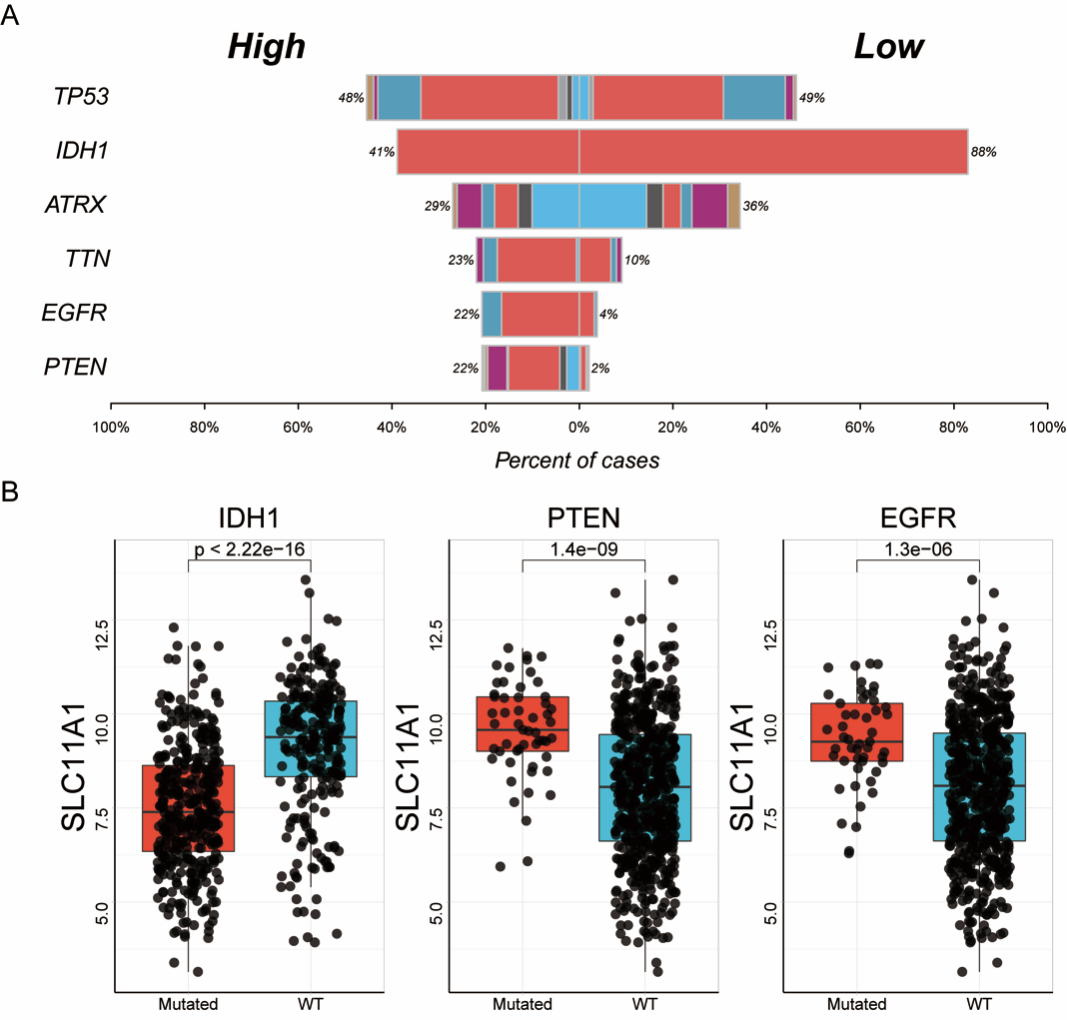
**

**Supplementary Figure 1. SLC11A1 associated with a variety of important mutations in gliomas.**

(A) coBarplot showed that high SLC11A1 subgroup was more likely to have EGFR and PTEN mutations compared with low subgroup. (B) Expression of SLC11A1 in context of different mutations of gliomas.


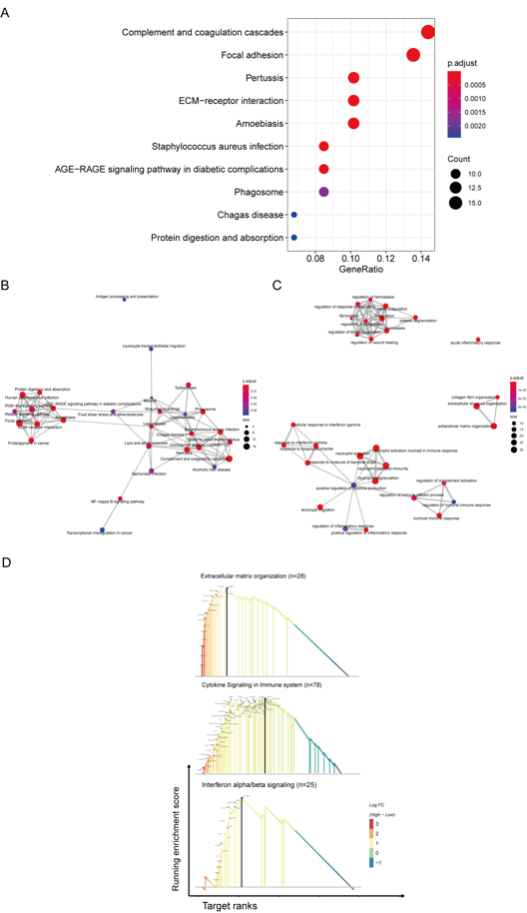


**Supplementary Figure 2. Biological annotations of DEGs.**

(A) KEGG analysis for differential expression genes between high and low SLC11A1 subgroups. The X-axis represents gene ratio and the Y-axis represents different enriched pathways. (B) Enrichment Map of GO terms. (C) Enrichment Map of KEGG terms. (D)Gene set enrichment analysis (GSEA) of DEGs.

**
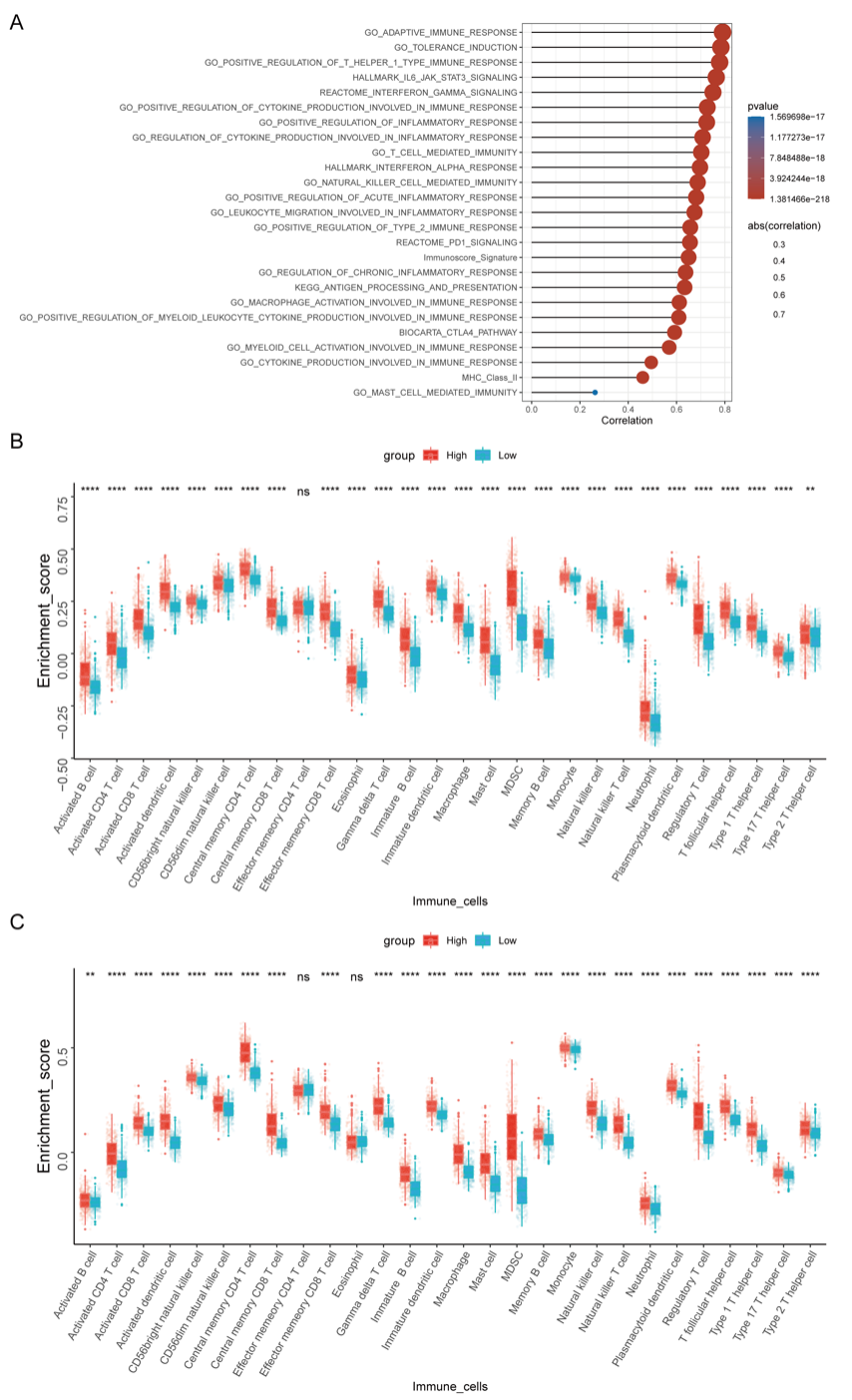
**

**Supplementary Figure 3. SLC11A1 expression correlates with glioma immune activation and microenvironment.**

(A) Dot-plot showing SLC11A1-associated GSVA scores of 25 innate and adaptive immunity-related gene sets. Sizes of dots represent the correlation. Boxplot showed relative abundance of 28 immune cells in gliomas, comparison between high- and low-SLC11A1 subgroups. (B) CGGA; (C) TCGA. *, P < 0.05; **, P < 0.01; ***, P < 0.001; ****, P < 0.0001, ns = no significance (Wilcoxon test).


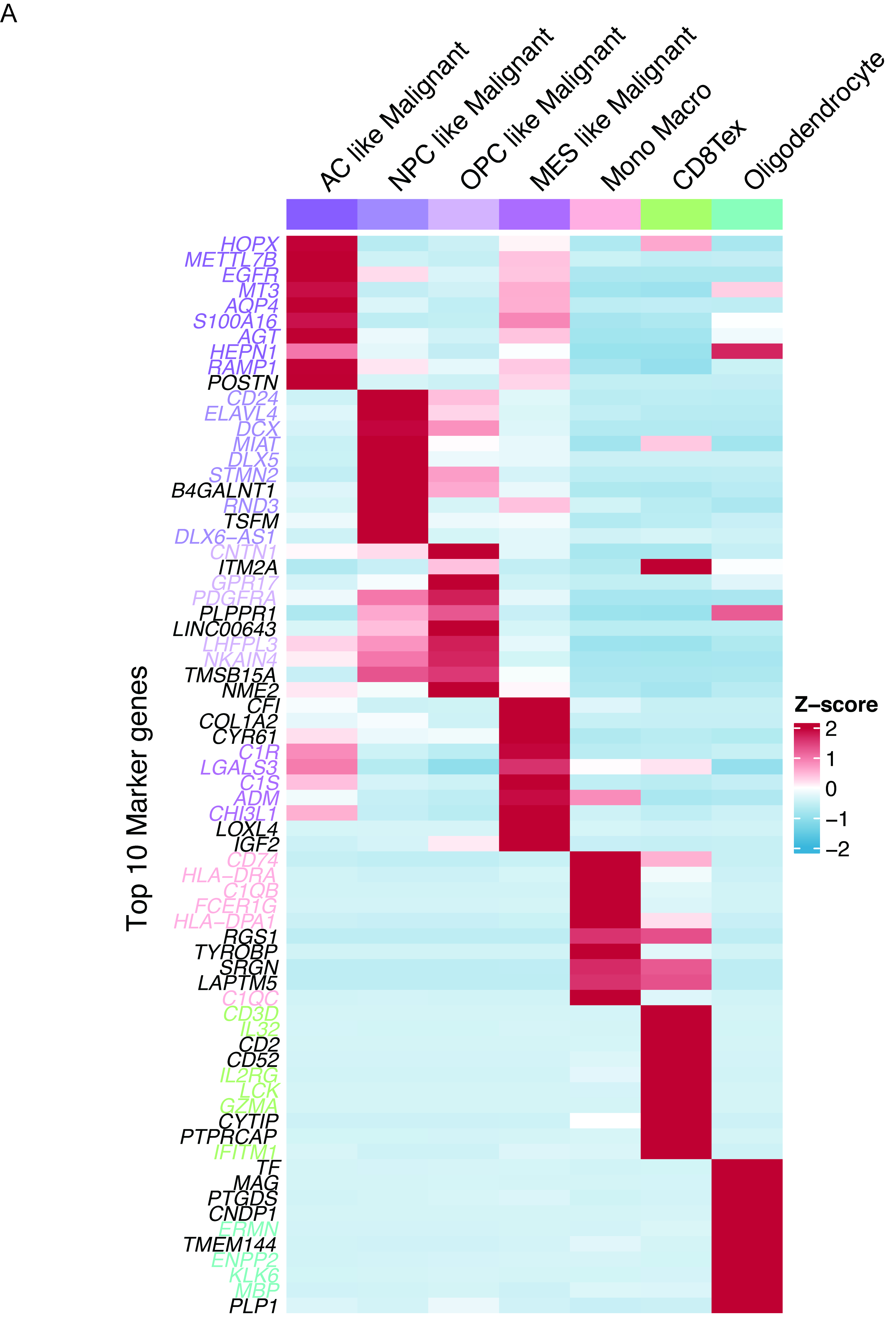


**Supplementary Figure 4. Marker genes of cell clusters from scRNA-seq.**

(A) Top 10 marker genes of each celltypes.


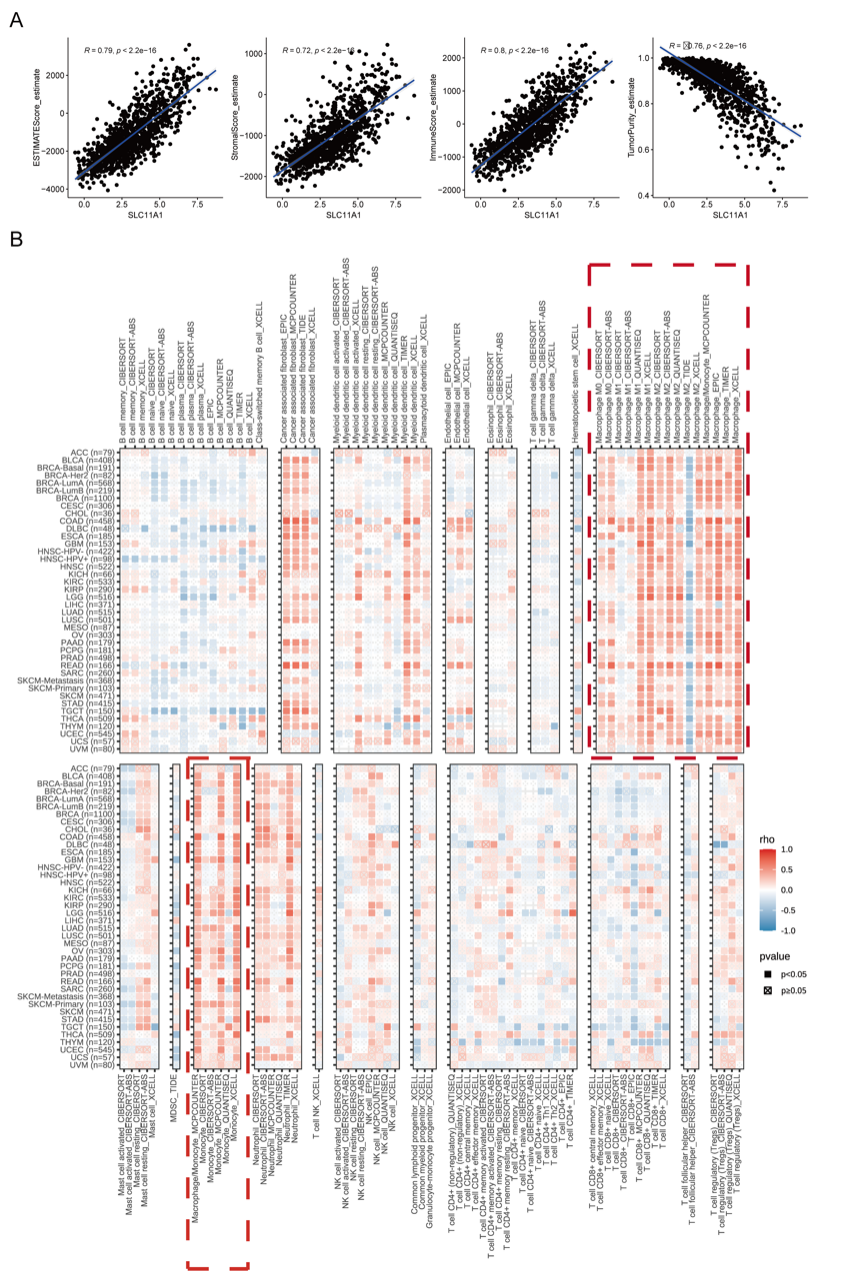


**Supplementary Figure 5. SLC11A1 implies the immune microenvironment of gliomas.**

(A) Correlation between the expression of SLC11A1 and ESTIMATE score. (B) Heatmap showing the strong correlation between SLC11A1 and immune cells, especially monocytes and macrophages, based on several different algorithms and pan-cancer expression data.


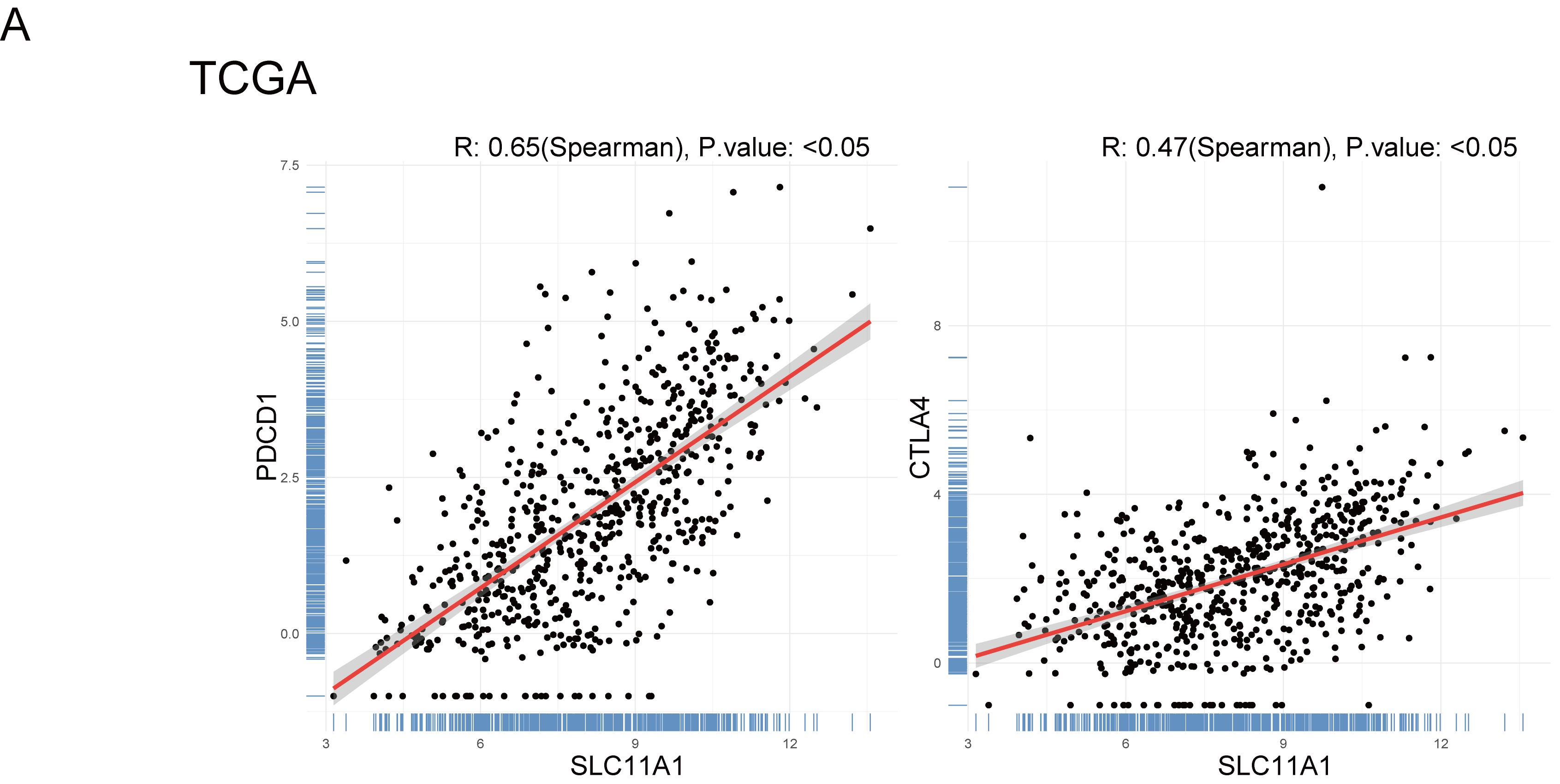


**Supplementary Figure 6. SLC11A1 as a good index for predicting patients’ sensitivity to glioma immunotherapy.**

(A) Correlation between SLC11A1 and PDCD1 and CTLA4 (TCGA)


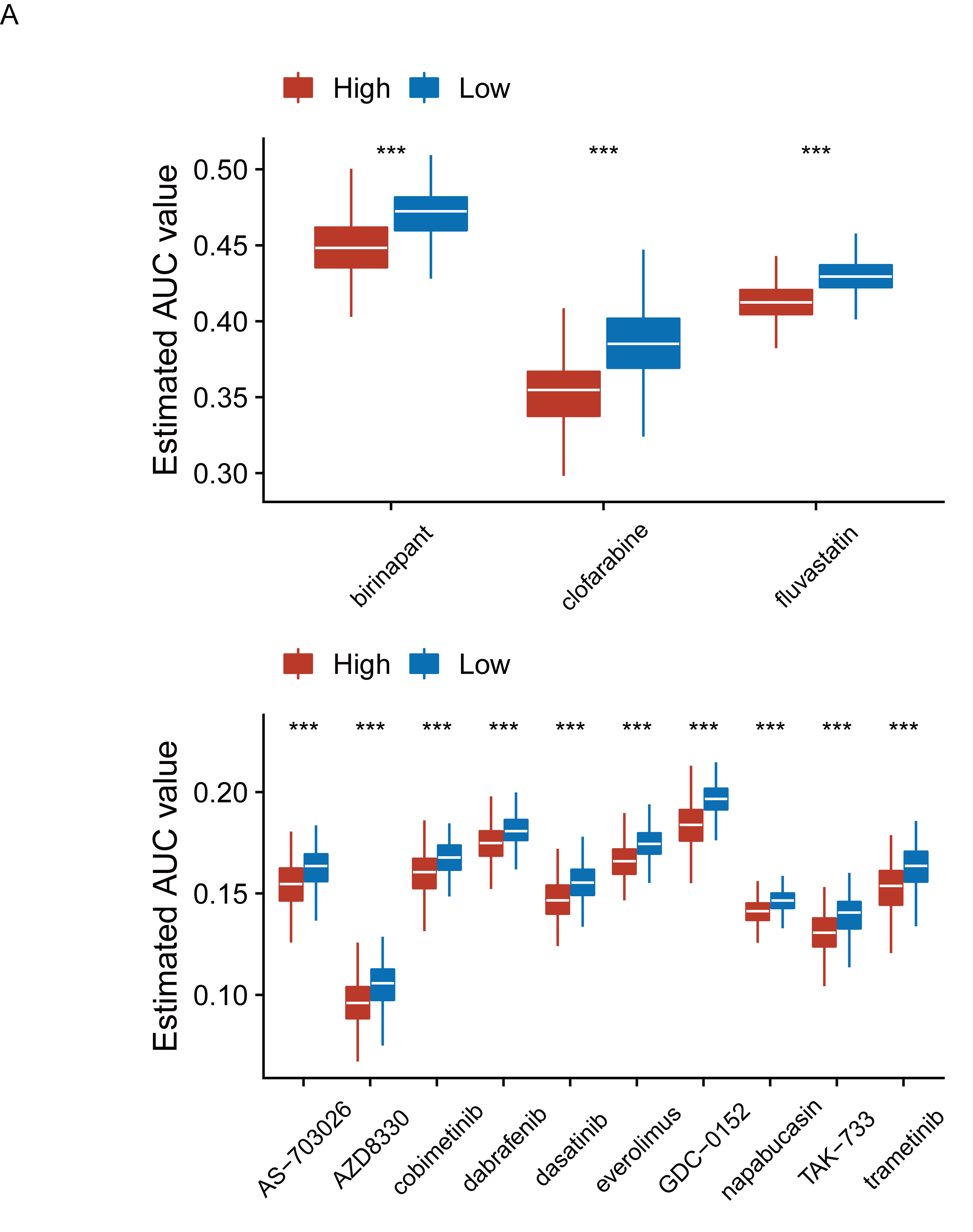


**Supplementary Figure 7. Identification of candidate agents with higher drug sensitivity in glioma patients.**

(A) Differential drug response analysis of three CTRP-derived compounds and ten PRISM-derived compounds. Note that lower values on the y-axis of boxplots imply greater drug sensitivity**.** *, P < 0.05; **, P < 0.01; ***, P < 0.001; ****, P < 0.0001, ns = no significance (Wilcoxon test).

**Supplementary Table 1. Datasets used in this study.**

**Supplementary Table 2. Marker genes of 25 immune-related pathways**

**Supplementary Table 3. Marker genes of TIS (T-cell inflammatory signature).**
